# Large Scale Gene Expression Meta-Analysis Reveals Tissue-Specific, Sex-Biased Gene Expression in Humans

**DOI:** 10.3389/fgene.2016.00183

**Published:** 2016-10-13

**Authors:** Benjamin T. Mayne, Tina Bianco-Miotto, Sam Buckberry, James Breen, Vicki Clifton, Cheryl Shoubridge, Claire T. Roberts

**Affiliations:** ^1^Robinson Research Institute, University of AdelaideAdelaide, SA, Australia; ^2^Adelaide Medical School, University of AdelaideAdelaide, SA, Australia; ^3^School of Agriculture, Food and Wine, Waite Research Institute, University of AdelaideAdelaide, SA, Australia; ^4^Harry Perkins Institute of Medical Research, The University of Western AustraliaPerth, WA, Australia; ^5^Plant Energy Biology, Australian Research Council Centre of Excellence, The University of Western AustraliaPerth, WA, Australia; ^6^Bioinformatics Hub, School of Biological Sciences, University of AdelaideAdelaide, SA, Australia; ^7^Mater Research Institute, University of QueenslandBrisbane, QLD, Australia

**Keywords:** sex-biased gene expression, meta-analysis, microarray, human, organs

## Abstract

The severity and prevalence of many diseases are known to differ between the sexes. Organ specific sex-biased gene expression may underpin these and other sexually dimorphic traits. To further our understanding of sex differences in transcriptional regulation, we performed meta-analyses of sex biased gene expression in multiple human tissues. We analyzed 22 publicly available human gene expression microarray data sets including over 2500 samples from 15 different tissues and 9 different organs. Briefly, by using an inverse-variance method we determined the effect size difference of gene expression between males and females. We found the greatest sex differences in gene expression in the brain, specifically in the anterior cingulate cortex, (1818 genes), followed by the heart (375 genes), kidney (224 genes), colon (218 genes), and thyroid (163 genes). More interestingly, we found different parts of the brain with varying numbers and identity of sex-biased genes, indicating that specific cortical regions may influence sexually dimorphic traits. The majority of sex-biased genes in other tissues such as the bladder, liver, lungs, and pancreas were on the sex chromosomes or involved in sex hormone production. On average in each tissue, 32% of autosomal genes that were expressed in a sex-biased fashion contained androgen or estrogen hormone response elements. Interestingly, across all tissues, we found approximately two-thirds of autosomal genes that were sex-biased were not under direct influence of sex hormones. To our knowledge this is the largest analysis of sex-biased gene expression in human tissues to date. We identified many sex-biased genes that were not under the direct influence of sex chromosome genes or sex hormones. These may provide targets for future development of sex-specific treatments for diseases.

## Introduction

Differences in both disease severity, prevalence, symptoms, and age of onset vary greatly between males and females (Morrow, 2015). For example, cardiovascular disease is one of the leading causes of death, affecting up to 55% of females but only 44% of males in Europe (Möller-Leimkühler, 2007). Sex differences are also evident in the risk factors for cardiovascular disease, such as diabetes which increases the risk for cardiovascular disease 2–3 fold in males but 3–7 fold in females (Eastwood and Doering, 2005). Sex differences have also been identified in the age of onset of brain diseases such as schizophrenia, where males develop symptoms between 18 and 25 years of age whereas females develop symptoms between 25 and 35 years (Ochoa et al., 2012). Moreover, reported atonic seizures in epilepsy are more frequent in males compared to females (6.5 vs. 1.7%; Carlson et al., 2014). These sex differences in diseases may be the result of tissue-specific differential gene expression between males and females. In schizophrenia, genes relating to energy metabolism have been found to have altered expression in the prefrontal cortex of only males (Qin et al., 2016). Therefore, gene expression may have a role in orchestrating sex differences in the prevalence of diseases.

Many studies neglect to account for sample sex in the design and analysis of their experiments (Mogil and Chanda, 2005; Beery and Zucker, 2011). Historically, females have been excluded from biomedical studies, due to the assumption that their hormonal cycles are a confounding factor in experimental manipulations (Zucker and Beery, 2010; Beery and Zucker, 2011). Despite females and males sharing highly similar genomes, there are numerous sex-specific traits in phenotype, physiology, and pathology. Sexually dimorphic traits can be influenced by sex chromosome genes or sex hormones, but may extend beyond these influences. Sex differences may arise through alterations in autosomal gene regulation but the true extent of sex specific differential gene regulation is not fully known. Understanding these differences may dictate that future research should consider sex as a biological confounder (Zucker and Beery, 2010). Sex differences in many traits are often small and require large sample sizes for studies to be sufficiently powered. The substantial increase in the number of large publicly available genomic data sets could assist in determining the true extent of sex-biased gene expression but to date there are no large-scale meta-analyses investigating this in adult human tissues.

Previous studies have reported sex-biased gene expression in the human brain (Vawter et al., 2004; Reinius and Jazin, 2009; Weickert et al., 2009; Kang et al., 2011; Trabzuni et al., 2013), pancreas (Hall et al., 2014), heart (Fermin et al., 2008), and liver (Zhang et al., 2011). Most studies identify sex-biased genes as those located on the sex chromosomes and it is well-known that these are a source of differentially expressed genes between the sexes (Carrel and Willard, 2005). In mammalian, female, somatic cells, one X chromosome is randomly inactivated by a process referred to as X chromosome inactivation (XCI; Carrel and Willard, 2005; Yang et al., 2010). In normal human XX females, up to 15% of genes on the X chromosome escape XCI, unlike the case in mice where very few escape inactivation (Carrel and Willard, 2005; Yang et al., 2010). Escape from XCI results in a number of genes that are expressed more highly in females compared to males. In addition, autosomal genes have also been shown to be sex-biased in human tissues including the brain (Trabzuni et al., 2013), heart (Fermin et al., 2008) and placenta (Buckberry et al., 2014b). Furthermore, sex differences in the brain in diseases such as multiple sclerosis (MS) are related to autosomal genes and are not regulated by sex chromosome genes (Voskuhl and Palaszynski, 2001; Ebers et al., 2004). These studies highlight the importance of investigating sex differences outside the context of reproductive and sex chromosome factors. In order to characterize the true extent of sex-biased gene expression in humans, we performed a large meta-analysis of publicly available microarray data. We limited our analysis to tissue samples from healthy individuals, reducing the possible effect that diseases may have on gene expression. Our analysis revealed consistencies in sex differences that are widespread in a range of human tissues. Furthermore, we have identified sex-biased genes that are disease-related, suggesting possible mechanisms for the associations of sex with an increased risk of certain diseases.

## Materials and methods

### Data collection

Data sets were from different microarray platforms and therefore pre-processing was tailored to each platform. Briefly, data from Illumina platforms were pre-processed using Beadarray prior to quantile normalization (Dunning et al., 2007). Data from Affymetrix platforms were pre-processed and quantile normalized using the robust multiarray average (RMA) or GeneChip-RMA (GC-RMA) where appropriate that is implemented in Simpleaffy (Wilson and Miller, 2005). Batch effects in data sets were corrected for using the “combat” function in the SVA package (Leek et al., 2012). Outliers were identified and removed using ArrayQualityMetrics by analysing MA plots (Kauffmann et al., 2009).

### Sample sex identification

To identify sample sex in each data set we used the massiR Bioconductor package (Buckberry et al., 2014a). This R package uses unsupervised clustering of probes that target Y chromosome genes to identify sample sex. In data sets where sample sex was supplied, we found an agreement in all predicted and supplied sample sex identification.

### Differential gene expression analysis

Probes were re-annotated to Ensembl gene identifiers using biomaRt (Durinck et al., 2009). In tissues where only one data set was found to be useable, sex-biased gene expression was determined using the Empirical Bayes methods within limma (Ritchie et al., 2015). For tissues that were present in >1 data set, differential gene expression analysis was performed using the *meta*GEM package (https://spiral.imperial.ac.uk/handle/10044/1/4217) and using the inverse–variance method as previously described (Ramasamy et al., 2008). For each probe, study specific effect sizes were calculated, by determining the mean and standard deviation for each probe which was corrected using Hedges' g (accounts for the number of samples in each dataset). *Z* statistics were calculated for each gene identifier which was used to calculate a nominal *p*-value to give a corrected *p*-value (false discovery rate, FDR).

### Androgen and estrogen response elements

To determine which genes contained androgen response elements (AREs), we firstly downloaded the coordinates of AREs from JASPAR (Hu et al., 2010; Mathelier et al., 2014) and determined the positions within the genome in relation to genes and genomic locations. This was performed using the matchGenes function in the bumphunter Bioconductor package (Jaffe et al., 2012) and UCSC hg19 annotation package (BP)^1^. For estrogen response elements (EREs) we used a previous study that lists genes that are targets of ERα (Jin et al., 2004).

### Identifying enriched transcription factors

Transcription factor (TF) binding sites within 10 kb upstream/downstream of sex-biased genes were analyzed using oPOSSUM-3 and the JASPAR vertebrate core profiles (Kwon et al., 2012; Mathelier et al., 2014). We chose 10 kb upstream/downstream of genes as this was the largest range the oPOSSUM-3 would allow. Thus, we sought to identify all possible TF binding sites enriched within sex-biased genes. For each sex-biased gene in each tissue, the TF binding site motifs were searched with a conservation cut-off of 0.4, an 85% threshold for the matrix score and minimum specificity of 8 bits. The resulting TF analysis was limited to the most enriched TFs which were defined as those with the highest Fisher's exact test and *z*-score rankings.

### Gene ontology

Gene ontology (GO) analysis was performed using all human genes in the Database for Annotation, Visualization, and Integrated Discovery (DAVID) v6.7 (Huang da et al., 2009) and g:Profiler (Reimand et al., 2016). GO terms were considered significant if the corrected *p*-value (FDR) < 0.05.

A more detailed account of the methodology is provided in File S1.

## Results and discussion

### Overview of publicly available microarray data

Using the Gene Expression Omnibus (GEO; Barrett et al., 2013) and ArrayExpress (Brazma et al., 2003) we identified 22 microarray data sets containing a total of 2502 samples, in 15 different human tissues (Table 1). We excluded pooled samples and limited our analyses to data sets with >10 samples to allow better determination of sample sex. To increase the number of useable data sets we used massiR (Buckberry et al., 2014a) to identify and to verify the sample sex in all data sets. From the 22 chosen studies, 10 had sample sex metadata and within these we found concordance with all the predicted and supplied sample sex information. Female samples (*N* = 803) made up 32% of all samples across all data sets (Table 1).

**Table 1:** **Gene expression data involving 15 healthy tissues**.

**Organ/tissue**	**GEO accession**	**Microarray manufacturer**	**Samples in data set**	**Control samples**	**Sample after pre-processing**	**Males**	**Females**
Bladder	GSE13507	Affymetrix	256	68	68	48	20
Brain	GSE45642	Affymetrix	670	670	659	493	166
Brain	GSE11512	Affymetrix	80	44	44	29	15
Brain	GSE54572	Affymetrix	24	12	12	5	7
Brain	GSE36192	Illumina	911	911	911	622	289
Brain	GSE44456	Affymetrix	39	39	39	28	11
Colon	GSE8671	Affymetrix	62	25	23	15	8
Colon	GSE41328	Affymetrix	20	10	10	8	2
Heart	GSE55231	Illumina	129	129	118	69	49
Heart	GSE26887	Affymetrix	24	24	23	19	4
Heart	GSE57338	Affymetrix	313	136	136	97	39
Kidney	GSE43974	Illumina	554	118	118	73	45
Kidney	GSE50892	Affymetrix	17	17	15	9	6
Liver	GSE61276	Illumina	106	50	48	22	26
Liver	GSE23649	Illumina	69	69	68	42	26
Liver	GSE38941	Affymetrix	27	10	10	4	6
Lung	GSE10072	Affymetrix	107	49	46	32	14
Lung	GSE18995	Affymetrix	35	35	34	15	19
Lung	GSE51024	Affymetrix	96	41	39	34	5
Pancreas	GSE15471	Affymetrix	78	36	35	19	16
Thyroid	GSE33630	Affymetrix	105	45	35	10	25
Thyroid	GSE65144	Affymetrix	25	13	12	7	5
		Total	3747	2551	2502	1699	803

*Each row corresponds to a data set where only healthy tissue was used within this analysis. The columns report the Microarray manufacturer, total number of samples, and which data sets supplied sample sex*.

**Figure 1:**
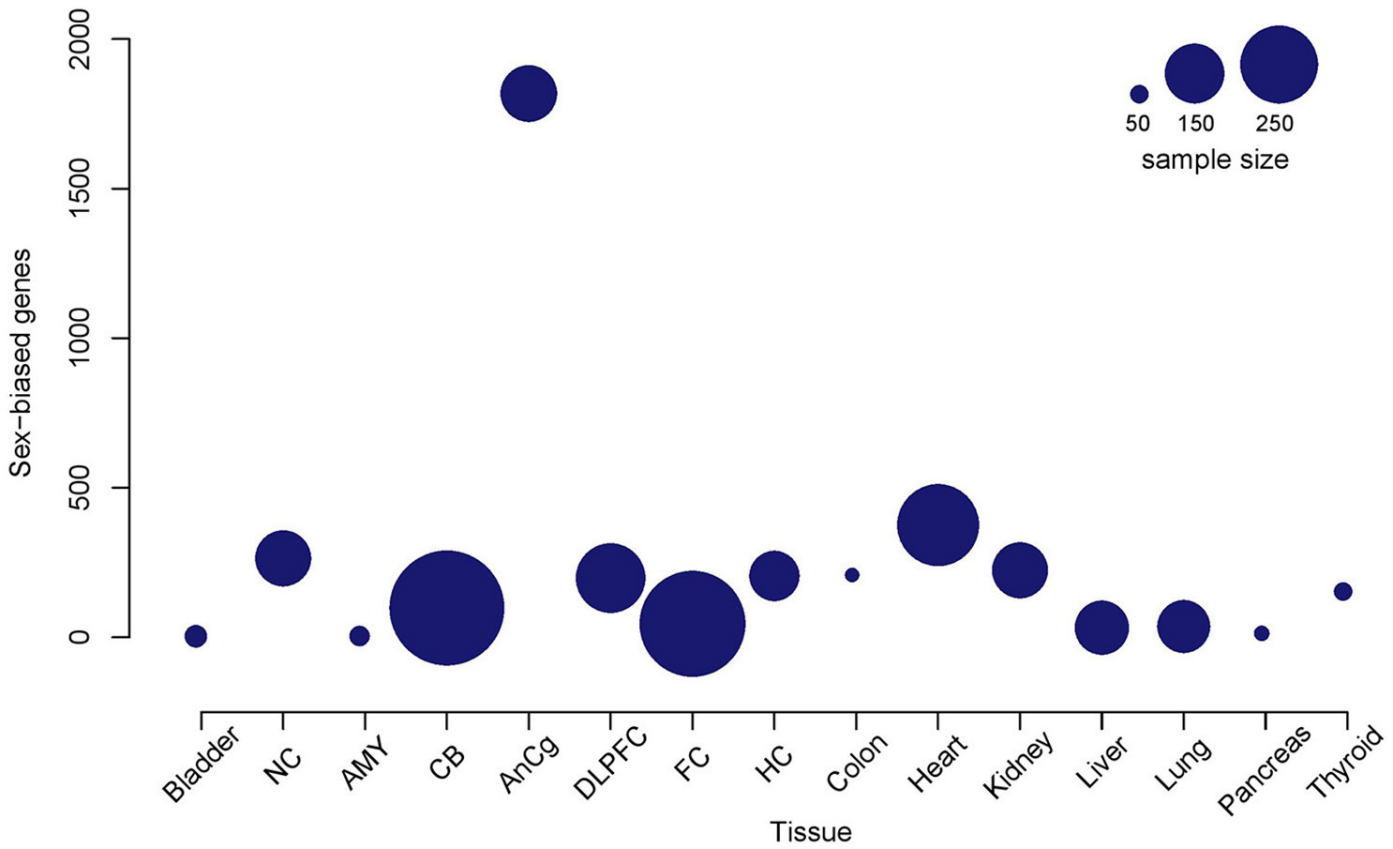
**Total number of detectable sex-biased genes relative to the sample size in each tissue**. A bubble plot of each tissue where the size of the bubble is proportional to the sample size of the tissue. Bubbles that are higher on the y-axis are tissues that demonstrate a higher number of detectable sex-biased genes. Nucleus accumbens (NC); amygdala (AMY); cerebellum (CB); anterior cingulate cortex (AnCg); dorsolateral frontal cortex (DLPFC); frontal cortex (FC); hippocampus (HC).

Sex differences in autosomal gene expression are typically small so in order to increase statistical robustness, we performed multiple testing corrections in three different analyses for each tissue. We determined the adjusted *p*-value implemented by Benjamini and Hochberg (1995) for each autosomal gene, where (1) all the chromosomes were included, (2) the Y chromosome was excluded, and (3) both the X and Y chromosomes were excluded in the analysis (Table 2). In general, we observed a reduction in the number of autosomal genes that were significantly sex-biased when we removed sex chromosomes from the analysis. Since most genes located on the sex chromosomes had the smallest adjusted *p*-value, their removal from the analysis slightly increased the adjusted *p*-value for all other genes. Here we supply the adjusted *p*-values for all three analyses (Tables S1– S3) but discuss only autosomal genes that were significantly different in all three cases. Furthermore, the sample size in each tissue was not reflective of the total number of genes differentially expressed between males and females (Figure 1). For example, despite the frontal lobe of the cerebral cortex or frontal cortex (FC) and cerebellum (CB) data sets containing the greatest number of samples, with 455 and 553 samples, respectively, we detected only a small number of sex-biased genes compared to other tissues such as the anterior cingulate cortex (AnCg) and the heart which contained the greatest number of sex-biased genes with average sample sizes (Figure 1, Table 2).

**Table 2:** **Total number of sex-biased genes in each tissue**.

**Organ/tissue**	**No. of sex-biased genes (All chromosomes)**	**No. of autosomal sex-biased genes (Sex chromosomes included in analysis)**	**No. of autosomal sex-biased genes (Sex chromosomes removed)**	**No. of autosomal sex-biased genes (Y chromosome removed)**
Bladder	16	0	0	0
Brain (Nucleus Accumbens)	264	239	216	244
Brain (Amygdala)	17	6	0	0
Brain (Cerebellum)	98	59	45	52
Brain (Anterior Cingulate Cortex)	1818	1726	1690	1728
Brain (Dorsolateral Prefrontal Cortex)	198	180	165	169
Brain (Frontal Cortex)	45	10	27	7
Brain (Hippocampus)	205	183	174	180
Colon	218	199	162	190
Heart	375	348	334	346
Kidney	224	196	194	194
Liver	32	21	16	28
Lung	36	14	2	12
Pancreas	22	0	0	0
Thyroid	163	151	133	135

*Each column corresponds to the total number of genes that were differentially expressed between males and females in each analysis*.

### Sex-biased gene expression in the human brain

**Figure 2:**
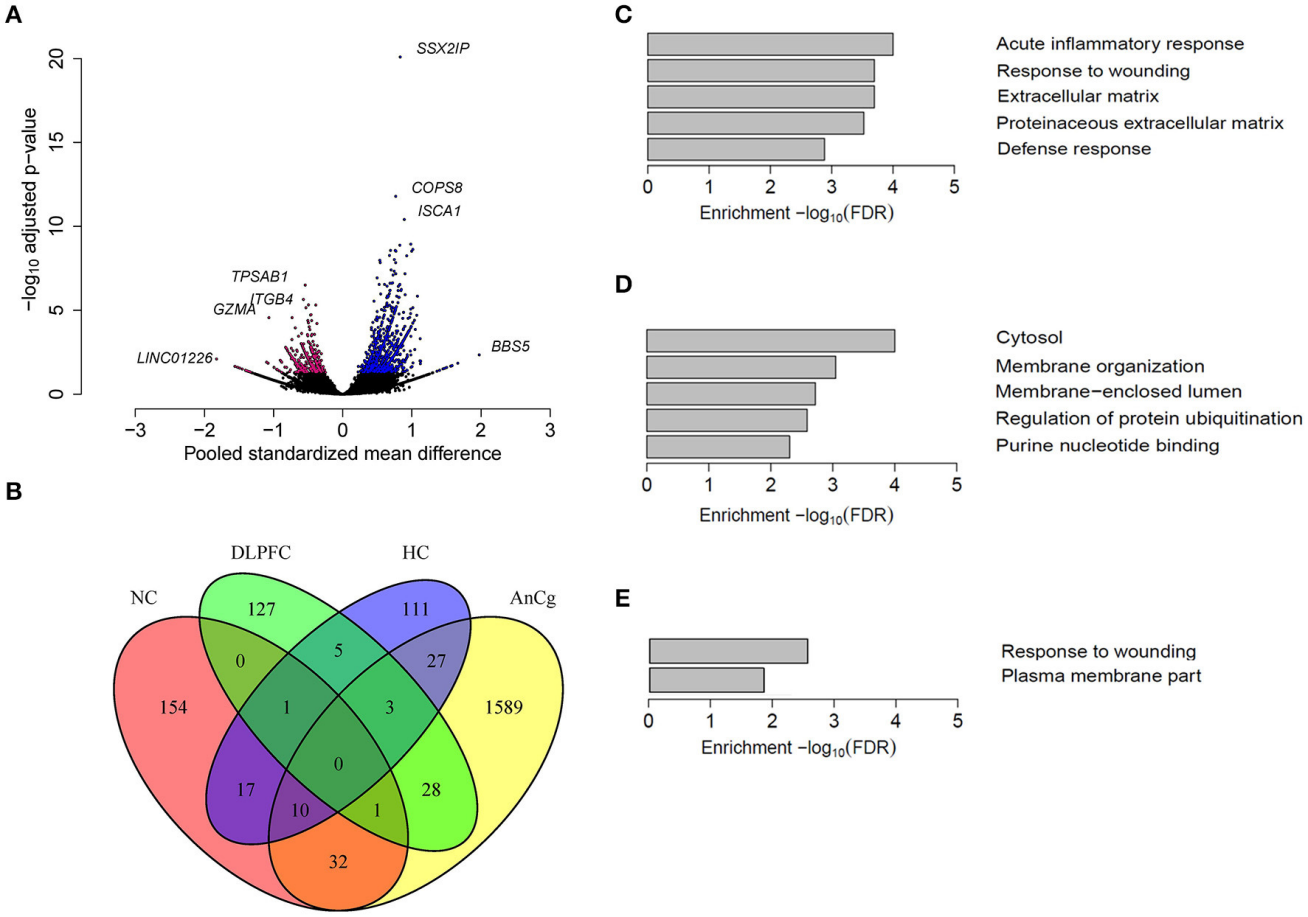
**Sex differences in autosomal gene expression in the human brain. (A)** A volcano plot representing the autosomal genes that were sex-biased in the AnCg. Pink colored dots represent genes that were significantly expressed more highly in females and blue colored dots represent genes that were expressed more highly in males. **(B)** A four-way venn diagram showing the overlap of sex-biased autosomal gene expression in different regions of the human brain. Most genes that were found to be sex-biased in one region were not sex-biased in another region. The top GO terms that were enriched for sex-biased genes in **(C)** Nucleus accumbens (NC), **(D)** anterior cingulate cortex (AnCg) and **(E)** hippocampus (HC).

Previous studies have found sex-biased gene expression in the human brain (Vawter et al., 2004; Reinius and Jazin, 2009; Weickert et al., 2009; Kang et al., 2011). We identified five data sets for seven brain regions and our analyses showed that each region had different numbers of differentially expressed genes (Tables 1, 2). Our findings were consistent with previous studies (Reinius and Jazin, 2009; Weickert et al., 2009; Kang et al., 2011), whereby the most striking differences in gene expression between the sexes were sex chromosome genes. These comprised most of the sex-biased genes in the amygdala (65%; AMY) and FC (78%). However, a large proportion of sex-biased genes were autosomal in the nucleus accumbens (91%; NC), AnCg (95%), dorsolateral prefrontal cortex (91%; DLPFC), CB (60%) and the hippocampus (89%; HC). Of the 1690 autosomal sex-biased genes in AnCg, 65% were expressed more highly in males (Figure 2A, Tables S1–S3). Conversely, we observed a greater proportion of autosomal genes expressed more highly in females in the NC (75%), DLPFC (68%), and the HC (62%). We also found that each brain region was unique in its proportion of sex-biased genes, with as many sex-biased genes in one brain region that were not sex-biased in another (Figure 2B).

An increase in the expression of heat shock proteins (HSPs) has been shown to have protective roles in pro-inflammatory responses (Grundtman et al., 2011). Consistent with a previous study (Lin et al., 2011), we found genes that encode for HSPs to have sex-biased expression in the human brain. Our analyses also identified genes that are involved in pro-inflammatory responses, such as those encoding interleukins, that are more highly expressed in females in NC, AnCg, DLPFC, and HC tissues (Tables S1–S3). By contrast, genes expressed more highly in males within the brain were related to energy production and growth, including ATPase's and insulin-like growth factors in the HC and NC, respectively, and *GAPDH* in the AnCg. We found sex-biased genes in the NC, AnCg, and HC to be enriched for GO as defined by DAVID v6.7 for terms relating to cellular functions, the immune response and energy production (Figures 2C–E, Table S4). We also used g:Profiler (Reimand et al., 2016) for a comparison of GO terms and found similar results to what was found by DAVID v6.7. For example, in the NC, AnCg, and HC we found that the gene upregulated in females were enriched for those involved in the immune response (GO:0006955). Whereas, genes upregulated in males were found to be enriched for GO terms such as generation of precursor metabolites and energy (GO:0006091). Overall, varying proportions and types of sex-biased genes were identified within different locations of the brain, suggesting that specific cortical regions may influence sexually dimorphic traits. As mentioned above, the AnCg contained the largest number of genes differentially expressed between males and females. The AnCg is one of the most recently evolved parts of the mammalian brain (Allman et al., 2001) and also has been shown to regulate behavior and act in a sex-specific manner (Liu et al., 2012). Furthermore, previous studies have identified sex differences in mood disorders and the AnCg is known to have a role in regulating mood (Seney and Sibille, 2014; Yang et al., 2015). In mice, the AnCg has also been shown to have a critical role in sexual interest of males for females (Wu et al., 2009) and hence the large number of genes that were differentially expressed between sexes in the AnCg may assist in the explanation for sexual dimorphism in behavior.

Sex biased gene expression in the brain may potentially contribute to differences in certain neurological diseases between sexes, such as the previously mentioned epilepsy. Sex differences in gene expression may mediate these differences in susceptibility or comprise part of the mechanistic pathways involved in their pathology. Previously, sex biased gene expression in the brain has been proposed to underlie the sex differences in schizophrenia (Trabzuni et al., 2013) which has an incidence of 1.4:1 between males and females (Abel et al., 2010). We found several genes that have been associated with brain disorders to be sex-biased within specific locations of the brain. For example in the AnCg, *NOTCH3*, a gene associated with hereditary stroke disorder (Joutel et al., 1996), and *ALDH3B1*, a gene associated with schizophrenia (Wang et al., 2009), were more highly expressed in females than males. On the other hand, *KCNH3*, a gene associated with epilepsy (Zhang et al., 2010), *GABRB3*, a gene associated with schizophrenia (Huang et al., 2014), epilepsy (Gurba et al., 2012), and autism (Buxbaum et al., 2002), *SNCA*, a gene associated with Parkinson's disease (Wang et al., 2015), and *RGS4*, a gene associated with schizophrenia (Jönsson et al., 2012), were all expressed more highly in males. Recently, sex-biased gene expression has also been identified during developmental stages of the human brain (Shi et al., 2016). Furthermore, genes associated with schizophrenia have been found to be upregulated in male brains as opposed to females across different developmental stages (Shi et al., 2016). This demonstrates consistency in sex-biased genes within the human brain across different studies. Taken together, these findings suggest possible mechanisms by which sex-specific prevalence of brain disorders may occur.

### The heart and kidney show opposite trends in sex differences in gene expression

Most of the heart gene expression data used in this study are from individuals with an average age of 47 years and we observed many sex differences in expression of genes associated with heart disease. It has been reported in elderly individuals (>75 years), isolated systolic hypertension can be up to 14% more prevalent in females than males (Maas and Appelman, 2010). We found *SCN10A*, a gene associated with hypertrophic cardiomyopathy (Iio et al., 2015), and *KCNE1*, a gene associated with long-QT syndrome (Splawski et al., 2000), to be expressed more highly in hearts from females. Interestingly, 62% of the 334 autosomal sex-biased genes in the heart were expressed more highly in females. The distribution of sex-biased genes across all chromosomes in the heart was similar to that in a previous study (Fermin et al., 2008). However, we report a much smaller number of sex-biased genes in the heart [375 genes in 277 samples (Table 2, Table S1) compared to 1800 genes in 102 samples in that study (Fermin et al., 2008)].

**Figure 3:**
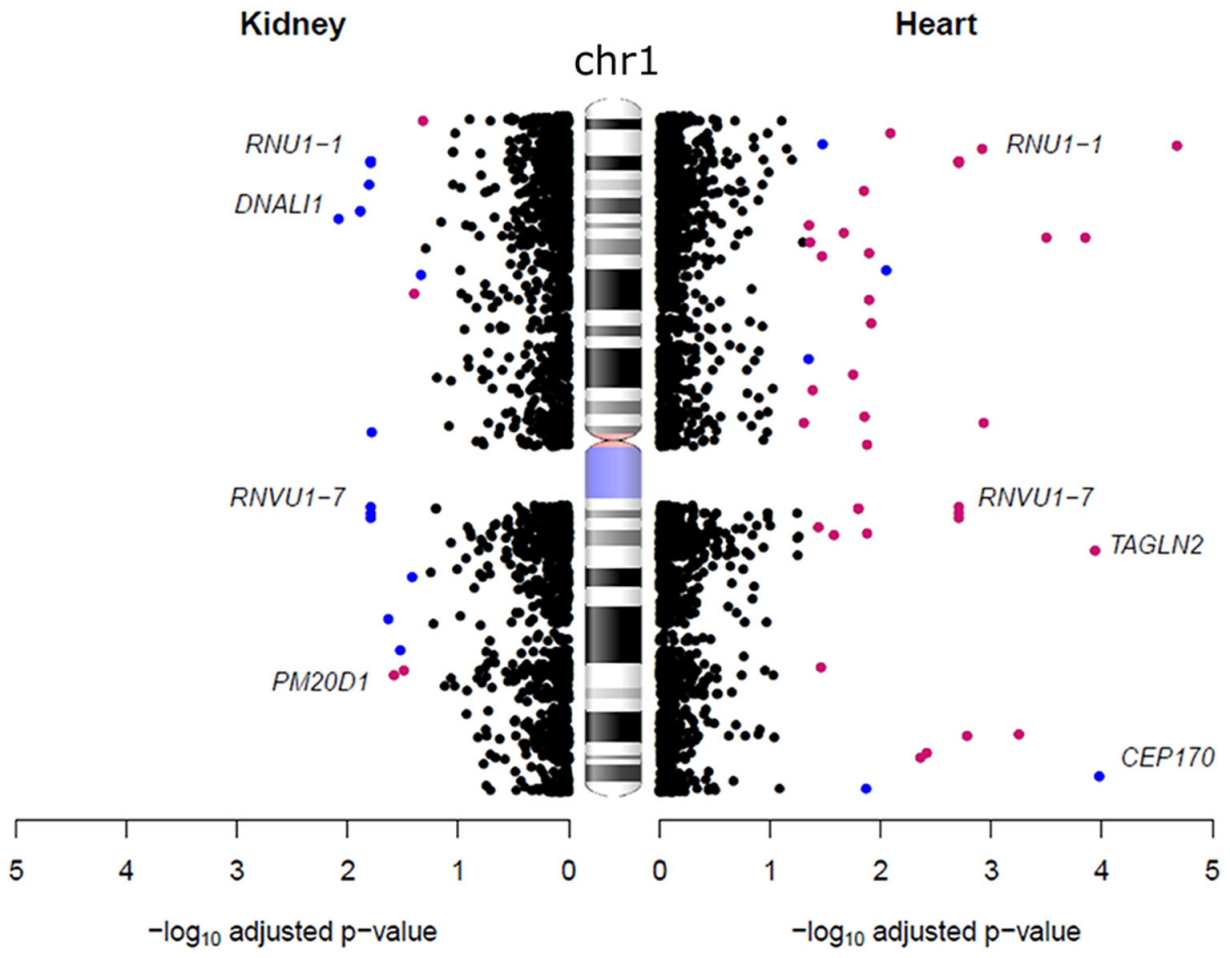
**Sex-biased gene expression differences on chromosome 1 in the heart and kidney**. Each dot represents a gene, blue dots are genes that were expressed more highly in males and pink dots are those expressed more highly in females. The ideogram of chromosome 1 was obtained from the (National Centre for Biotechnology Information, 2016) (NCBI).

Conversely, compared to the heart, we found an opposite trend in the kidneys, with 72% of a total of 194 autosomal genes being expressed more highly in males. We also identified six genes located on chromosome 1 that were expressed more highly in females in the heart that were more abundantly expressed in males in the kidney (Figure 3). These genes are from the RNA U1 family (*RNU1-1, RNU1-2, RNU1-3, RNU1-4, RNVU1-7*, and *RNU1-18*) that includes genes that regulate transcription, elongation and pre-mRNA splicing events (O'Reilly et al., 2013; Guiro and O'Reilly, 2015). It has been suggested that the expression of these genes is different between tissues to regulate organ specific alternative splicing events (Guiro and O'Reilly, 2015). Sex differences in alternative splicing have also previously been detected in the brain, where it has been found to affect 2.5% of expressed genes (Trabzuni et al., 2013). Apart from RNA U1 family all other sex-biased genes were only found to be expressed more highly in one sex.

### Sex hormones and gene expression

Many of the sex-biased genes we identified encode enzymes that are known to regulate the production of sex hormones. In the AnCg, three genes from the sulfotransferase family that regulates sulfate conjugation in estrogen precursors (Adjei et al., 2003; *SULT2A1, SULT1B1*, and *SULT1C1*) were expressed more highly in females. In addition, we also found *STS* [a gene involved in the production of estrogen precursors (Miki et al., 2002)] to be expressed more highly in females in the FC and CB, as well as in the heart and lung. We did not find any major sex differences in gene expression in the bladder, liver, lung, or pancreas, apart from genes located on the sex chromosomes and those that are involved in sex hormone production. This can be contradictory to that which has been found in mouse studies where thousands of genes have been found to be sex-biased (Yang et al., 2006; van Nas et al., 2009). This may reflect an evolutionary difference between the species. Apart from the brain, we found the largest number of sex-biased gene expression differences in the heart, kidney, colon, and thyroid (Table 2). Thyroid hormones are known to regulate sex hormone-binding globulin (SHBG) production, which transports androgens and estrogens through the bloodstream (Selva and Hammond, 2009). In the thyroid, 133 autosomal genes were sex-biased, 75% of which were expressed more highly in males. Genes that encode for growth factors and signaling molecules were highly expressed in the thyroid of males, such as *CCL28*, a growth factor in hematopoietic stem cells (Karlsson et al., 2013), *CMTM4*, a chemokine that regulates the cell cycle (Plate et al., 2010), and *GH1*, a gene that encodes for growth hormone (Vakili et al., 2014). These findings suggest a functional role for the thyroid in influencing sexually dimorphic traits such as metabolism, as well as sex differences in thyroid hormone secretion (Ehrenkranz et al., 2015). There is also evidence to suggest that thyroid hormones significantly influence testosterone levels (Meikle, 2004).

To determine if the differentially expressed genes between sexes were regulated by sex hormones, we quantified the number of genes that contained either AREs or EREs. For AREs we downloaded the coordinates of AR binding sites from the JASPAR database (Hu et al., 2010; Mathelier et al., 2014) and for EREs we used a list of previously reported ERα targets (Jin et al., 2004). In total, we identified 3014 different genes that were expressed more highly in either sex in at least one tissue. Of the 3014 genes, 875 contained AREs, 239 contained EREs and 86 contained both. On average 32% of autosomal genes that were sex biased in tissues contained AREs or EREs. Therefore, across all tissues analyzed approximately two-thirds of autosomal genes did not contain either AREs or EREs. Four hundred and eighty-nine genes contained AREs within gene bodies such as introns and exons, 216 genes contained AREs upstream and within the promoters, and 170 genes contained AREs located downstream of the gene. The precise locations of EREs were unknown as we were using a list of previously defined ERα targets. GO enrichment for genes that contained both AREs and EREs in each individual tissue did not produce any significant enrichment, most likely due to the lists of genes being too small. We therefore found it advantageous to combine the list of genes across different tissues since the list of genes in each tissue were too small to produce any significant results. The genes that contained either or both AREs or EREs and were expressed more highly in females were enriched for GO terms relating to response to wounding and inflammatory response. For example, we found genes related to interleukin signaling and inflammatory processes to be expressed more highly in females such as *TNFAIP6, IL10RB*, and *IFNA2* in the DLPFC, HC, and AnCg, respectively. On the other hand genes containing either or both AREs or EREs that were expressed more highly in males were enriched for GO terms relating to mitochondrion and generation of precursor metabolites and energy. As already mentioned, we found a variety of ATPase's to be expressed more highly in males in the AnCg, NC, DLPFC, CB, thyroid, colon, and kidney such as *ATP5G1, ATP6V1B2, ATP6V0B, ATP6V1C1*, and *ATP6V1A*. These results indicate that sex chromosome genes and sex hormones are key regulators of sex-biased gene expression across a range of tissues. However, our data also suggest a significant number of genes that have sex-biased expression may potentially be independent of direct influence by sex chromosomes or sex hormones.

### Sex-biased epigenetic modifications

Genes that are involved in the regulation of transcription and histone modifications also showed sex differences. In the colon, genes expressed more highly in males included those that encode for histones (*H3F3A, H3F3AP4, H3F3AP6*, and *H3F3BP1*) and ribosomal proteins (*RPS3A, RPS3AP26, RPS3AP6, RPL13A, RPL4, RPL4P4, RPL13AP5, RPS3AP5, RPS3AP47, RPL7A, RPL7AP6, RPL23AP74, RPL4P5, RPL3P4, RPL13AP20*, and *RPL13AP25*). These genes were also expressed more highly in males in other tissues such as the brain, heart, and kidney. It is worth mentioning that we also found other members of the RPL gene family to be more highly expressed in females in other tissues (Tables S1–S3). We also found sex bias in some genes that encode for enzymes that regulate histone modifications. For example, *SET*, a gene that inhibits nucleosome and histone H4 acetylation (Krajewski and Vassiliev, 2011) was expressed more highly in males in the DLPFC, *SMYD3*, a histone methyltransferase (Hamamoto et al., 2004), *PRMT2, PRMT5*, and *PRMT8* [histone arginine methyltransferases (Di Lorenzo and Bedford, 2011)] were more highly expressed in males in the AnCg and DLPFC (Tables S1–S3). Together these findings suggest that sex differences in tissue-specific gene expression extend from sex hormones and into genes that regulate gene expression and translation. Furthermore, our findings of sex bias in genes that encode for histones and histone modifying enzymes in most tissues suggest the possibility that sex-specific epigenetic modifications act on transcription that may result in phenotypic sex differences.

### X-linked sex-biased gene expression

**Figure 4:**
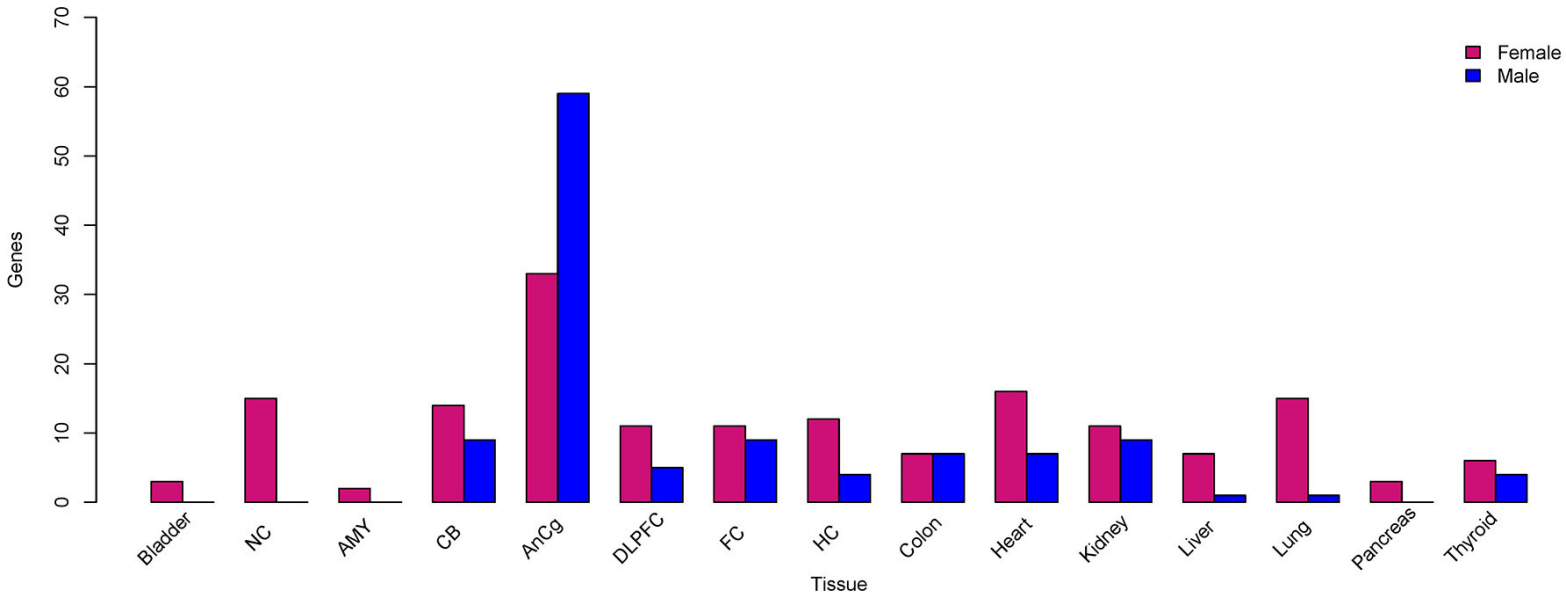
**X-linked sex-biased gene expression**. The total number of genes located on the X chromosome that were expressed more highly in females (pink) and males (blue) compared to the opposite sex, respectively.

**Figure 5:**
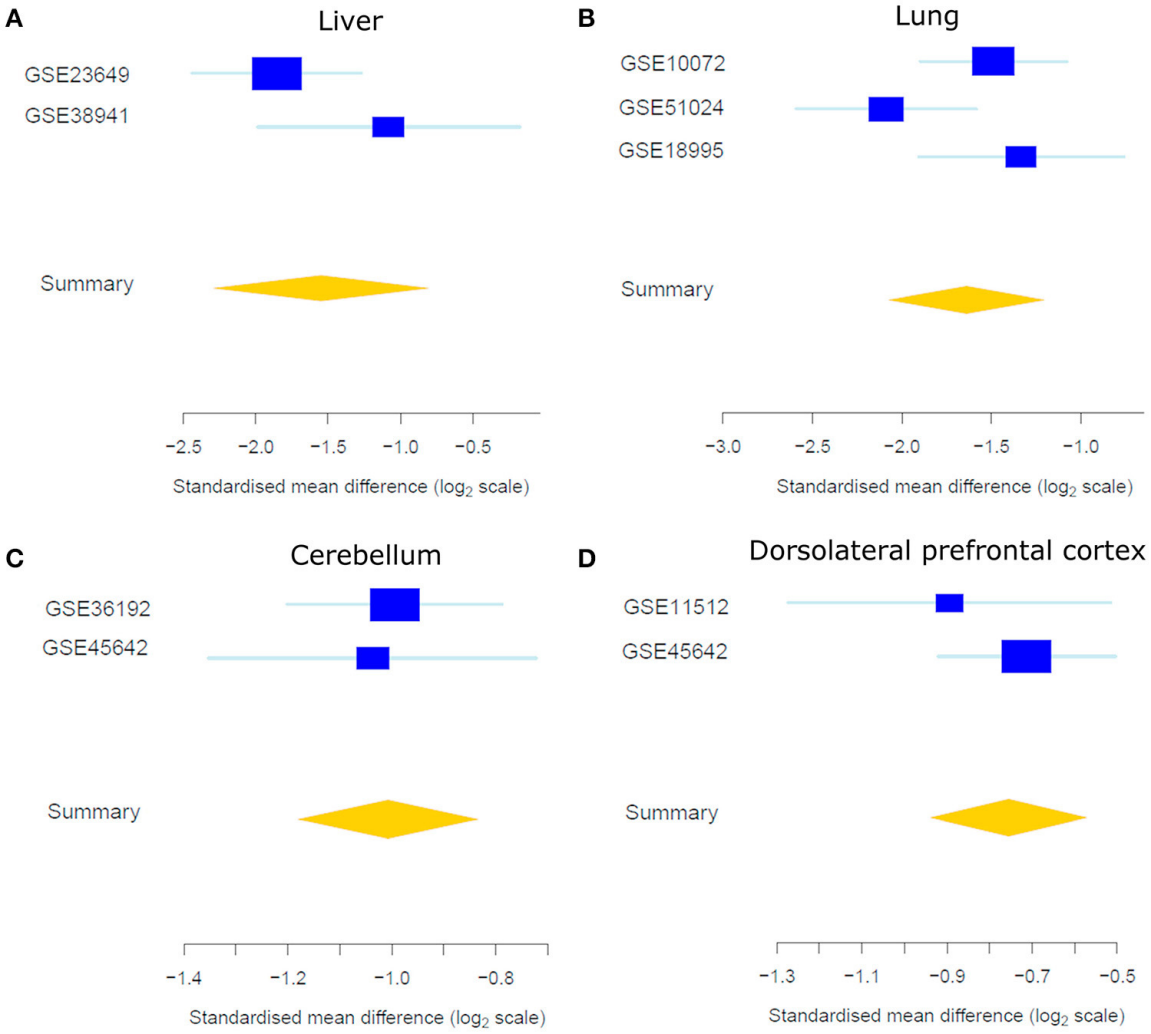
**Forest plots of the standardized mean difference of *KDM6A* expression showing higher expression in females in the liver (A), lung (B), CB (C), and DLPFC (D)**. Each blue box is representative of the study size in each data set and horizontal lines are standard error. The yellow diamond represents the overall gene summary for *KDM6A* in each tissue.

As expected, a majority of X-linked, sex-biased genes were expressed more highly in females (Figure 4), with the exception of those in the AnCg in which 75% were more abundantly expressed in males. The mechanism by which genes on the single copy X chromosome in males could be expressed more highly than in females with two copies is obviously likely to be associated with XCI but another mechanism is likely to be active and requires investigation. Although we do report Y chromosome genes in our analysis (Table 2, Table S1), we do not consider these genes as differentially expressed between sexes, since females do not have a Y chromosome. We do, however, consider the reported Y chromosome genes as detectable in the analyzed tissues and act as a positive control and these genes may have potential roles in the male phenotype in these tissues. Many X-linked genes that were expressed more highly in females have been previously reported to escape XCI (Cotton et al., 2015). Not surprisingly, we consistently found *XIST* and *JPX* [genes that orchestrate XCI (Augui et al., 2011; Lee, 2011)] to be expressed more highly in females and interestingly, many sex-biased X-linked genes that are known to regulate gene expression have been defined previously (Bellott et al., 2014). For example, we found *KDM6A* (Figure 5), a gene that regulates chromatin modifications, to be expressed more highly in females in the liver, lung, DLPFC, NC, AMY, FC, bladder, and CB. In addition, forest plots (Figure 5) demonstrate consistency between individual data sets of *KDM6A* expression showing higher expression in females across different tissues. Furthermore, we also found *KDM5C* to be expressed more highly in females in the lung, FC, bladder, and CB. Genes that are involved in post-transcriptional processes and more highly expressed in females in the liver, thyroid, FC, and CB, include *ZRSR2, DDX3X* which are involved in alternative splicing. In addition, we also found translation regulators *EIF1AX* and *RPS4X*, to be expressed more highly in females in the lung, pancreas, HC, and colon.

Across all tissues, we found a total of 86 different genes on the X chromosome to be more highly expressed in males in at least one tissue. Twenty-two of the 86 X chromosome genes more highly expressed in males have homologous counterparts on the Y chromosome and are located within pseudoautosomal region 1 (PAR1; Ross et al., 2005), which may explain the differences in expression. However, not all X chromosome genes that were expressed more highly in males were within PAR1 or had homologous Y chromosome counterparts, such as *SMARCA2*, an ATPase and chromatin re-modeler (Takeshima et al., 2015). These findings suggest that X-linked sex-biased genes may potentially regulate autosomal gene expression such as the possible case of *SMARCA2*, through epigenetic modifications and post-transcriptional processes.

### Enriched transcription factors

We next investigated which TFs were enriched in the sex-biased genes by running a TF binding site (TFBS) enrichment analysis using oPOSSUM-3 and the JASPAR core motifs (Kwon et al., 2012; Mathelier et al., 2014). Both the Sry-related HMG box (SOX) and the Forkhead-box (FOX) family of TFs were enriched within 10 kb of the transcription start site (TSS) of sex-biased genes across all tissues (Table S5). The SOX TFs are vital for sex determination (Huang et al., 2015) and the FOX TFs are essential for embryonic development and also have roles in regulating the immune system (Coffer and Burgering, 2004; Jackson et al., 2010; Lam et al., 2013). Sex chromosome derived TFs such as *ZFX* and *SRY* were also enriched within 10 kb of the TSS. We also found the androgen receptor (*AR*) as an enriched TF within the AMY, CB, FC, bladder, and lung. In addition, *HNF1A* and *HNF1B* were enriched in genes upregulated in both males and females within all tissues apart from the NC and DLPFC. *HNF1A* and *HNF1B* are homeobox TFs that are required for expression of specific liver genes (Shih et al., 2001). These findings reveal TFs that may have important roles in regulating sexually dimorphic gene expression such as *HNF1A* and *HNF1B*, which as mentioned earlier have only previously been shown to be required for expression of specific liver genes (Shih et al., 2001). However, the genes that encode for the majority of the TFs that were enriched within sex-biased genes were not themselves differentially expressed between the sexes. Although in this study, we focus on gene expression, TFs undergo more processing post-transcription and therefore their protein abundance within tissues may differ between sexes.

### Sex differences in other tissues

In this study, we have analyzed sex-biased gene expression in 15 human tissues. However, we must acknowledge other studies that have also analyzed sex-biased gene expression. One of the largest studies that has analyzed sex-biased gene expression is the Genotype-Tissue Expression (GTEx) project (Melé et al., 2015). The GTEx project has used RNA-seq to analyse gene expression in a variety of different human tissues which would give a broader comparison of gene expression differences between tissues. In comparison to GTEx (Melé et al., 2015) we have analyzed sex-biased gene expression in five of the same human tissues which is represented as a Venn diagram (Figure S1). We found an overlap of sex chromosome genes as being sex-biased between this study and GTEx. However, there were many genes that we found to be sex-biased that were not in GTEx (Melé et al., 2015). A possible explanation for the difference between studies is that in GTEx only samples from175 individuals were used (Melé et al., 2015) as opposed to over 2500 in this study which provides much greater statistical power compared to GTEx (Melé et al., 2015). In addition, GTEx also used RNA-seq and were therefore able to quantify the expression of genes for which no probes were available in the microarrays used in this study.

### Bias of male samples

To prevent any biases in our analyses we have performed differential gene expression in tissues from all publicly available data to our knowledge. However, since most studies neglect to account for samples sex (Mogil and Chanda, 2005; Beery and Zucker, 2011), we unfortunately had a ratio of 2.1:1 males to females on average across all tissues analyzed. Therefore, this in itself may create some biases in our analyses. Across all data sets (Table 1) the ratio of males to females was skewed toward males apart from one data set containing thyroid samples (GSE33630), where the ratio was 2.5 females for every male.

To determine if the ratio of males to females affects the differential expression analyses we conducted a 10-fold cross validation of the differential gene expression analyses in the tissue where the ratio of males to females was the greatest. The AMY gene expression data had a ratio of 4.5 males to every female. In this analysis we randomly removed male samples from the analysis to make the number of each sex the same and then assessed which genes were differentially expressed between males and females. We performed this analysis 10 times and then compared which genes were consistently identified as sex-biased to our original analysis where we did not sub-set any male samples. In the analysis with the sex chromosomes included we found the sex chromosome genes (*XIST, RPS4Y1, DDX3Y, KDM5D, USP9Y, EIF1AY*, and *TTTY15*) consistently classified as sex-biased in the 10-fold cross validation. However, in the original analysis we identified four autosomal genes to be sex-biased and upregulated in females (Table S1). However, these four autosomal genes were not found to be sex-biased in the 10-fold cross validation. By performing the 10-fold cross validation, we removed samples which would have decreased our statistical power and therefore increased the magnitude of the adjusted *p*-value which is what occurred. Therefore, caution should be taken when interpreting the results of genes that were found to be sex-biased with an adjusted *p*-value close to 0.05 and in tissues where there is a large ratio of males to females. However, this analysis does provide reassurance that the sex chromosome genes that were found to be sex-biased in the original analysis were not greatly affected by the bias in male samples.

## Strengths and limitations

While our analyses reveal many sex differences in gene expression within a variety of tissues, there are several limitations to this study. Firstly, most tissues (where age was provided) were from individuals who were post-reproductive age (average age = 47 years) which may not have captured the true extent of sex-biased gene expression that would otherwise be evident during early adulthood when sex hormones are at their peak production. Thus, using data from older individuals limited our ability to assess sex-biased gene expression in individuals of reproductive age. We also report a number of genes previously associated with diseases and disorders that were differentially expressed between sexes. RNA expression differences do not necessarily cause phenotypic variation, as there are multiple levels of gene and protein regulation that can occur post-transcription. Next-generation sequencing, as opposed to microarrays used in this study, would allow a more complete assessment of sex-dependent gene expression differences but there is currently more samples that have been analyzed using microarrays and therefore more statistical power can be achieved. Furthermore, on average, 64% of genes differentially expressed between sexes in each tissue had a magnitude log_2_FC < 1. Most genes that were found to be sex-biased do not have large log_2_FC apart from genes located on the sex chromosomes. In addition, most genes that were found to be sex-biased across all tissues had a magnitude log_2_FC < 1.5 (Table S7). Therefore, future studies would need to be adequately powered to replicate our findings. Despite these limitations, to our knowledge this is the largest analysis of sex differences in gene expression across a range of human tissues.

Despite the large amount of genomic data that was available for this study it was unfortunate not to consider clinical and lifestyle factors such as age, smoking status, sample heterogeneity and body mass index (BMI) which may potentially have an effect on gene expression. We were unable to correct for these potential confounding factors because, as detailed in Table S6, most studies provide little or no clinical information about the samples. Furthermore, only 32% of all the samples analyzed in this study were from females which may potentially create a bias for genes to be more highly expressed in males. However, by acknowledging this limitation we draw attention to the bias toward using only males in biomedical research. We therefore urge future research in all fields of biomedical science to use an equal sex ratio in study design.

## Conclusions

Our analyses have revealed substantial differences in the transcriptional landscape between sexes across a range of human organs and tissues and highlight possible mechanisms by which gene expression may contribute to sexually dimorphic traits. Improved understanding of these is fundamental to understanding diseases with different prevalence between the sexes. Our data show that sex differences in gene expression vary widely across different tissues. We identified a consistent trend for genes known to regulate the immune system to be more highly expressed in females and those involved in energy production and growth were more highly expressed in males. These may be the result of different evolutionary pressures between the sexes. The brain demonstrates the largest differences in sex-biased gene expression with several sex-biased genes associated with specific brain disorders, providing insight into possible mechanisms for the association of sex-specific prevalence of certain brain disorders.

Our findings also indicate that many sex biased genes within tissues are independent of sex chromosome genes or sex hormones. Approximately 32% of autosomal genes in each tissue contained an ARE or ERE, which suggests there are other mechanisms that underpin sex differences in gene expression. One potential mechanism is through epigenetic factors, such as chromatin modeling which has been suggested to have sex specific functional roles (Silkaitis and Lemos, 2014).

Finally, our data demonstrate why it is important to consider sex as a biological confounder in biomedical studies. Future studies should incorporate sex differences in their analyses which will help to provide new insights in health and disease. The sex-biased genes identified in this study provide a basis for determining the mechanism by which sexual dimorphism occurs and potential causal pathways for sexually biased disease susceptibility. More importantly however, they provide potential targets for novel sex specific treatments.

## Authors contributions

BM designed, conducted the study, analyzed and interpreted the data, and wrote the manuscript. SB conceived the initial part of the study and provided intellectual input into the manuscript. JB, TB, and CR were all involved in the study design, provided critical discussion and intellectual input into the manuscript. CS and VC provided critical discussion and intellectual input into the manuscript. All authors read and approved the final manuscript.

## Funding

This project was funded in part by a National Health and Medical Research Council of Australia (NHMRC) Project Grant (GNT1059120) awarded to CR, CS, VC, and TB. CR is supported by a NHMRC Senior Research Fellowship GNT1020749. CS is supported by an Australian Research Council Future Fellowship (FT120100086). VC is supported by a NHMRC Senior Research Fellowship GNT1041918. SB is supported by an NHMRC-ARC Dementia Research Development Fellowship Grant (APP1111206). BM is supported by an Australian Post-graduate Award.

### Conflict of interest statement

The authors declare that the research was conducted in the absence of any commercial or financial relationships that could be construed as a potential conflict of interest.

## Footnotes

^1^BP., C. M. a. M., TxDb.Hsapiens.UCSC.hg19.knownGene: Annotation package for TxDb object(s). R package version 3.2.2.

## Supplementary material

Table S1 **Differential gene expression analysis between males and females in each tissue, including the sex chromosomes**. A list of differentially expressed genes between sexes in each tissue with all the chromosomes included in the analysis. A fold change > 0 indicates the gene is expressed more highly in males and a fold change < 0 indicates the gene is expressed more highly in females.

Table S2 **Differential gene expression analysis results with genes on the Y chromosome removed from the analysis**. A list of sex-biased genes with the Y chromosome genes removed from the analysis. A fold change > 0 indicates the gene is expressed more highly in males and a fold change < 0 indicates the gene is expressed more highly in females.

Table S3 **Differential gene expression analysis results with genes on the X and Y chromosomes removed from the analysis**. A list of sex-biased genes with the sex chromosome genes removed from the analysis. A fold change > 0 indicates the gene is expressed more highly in males and a fold change < 0 indicates the gene is expressed more highly in females.

Table S4 **Gene ontology results**. This table lists all the GO terms that were found to be enriched within each tissue. Only significant GO terms were found for the NC, AnCg, and HC.

Table S5 **Transcription factors that were found to contain enriched motifs with 10 kb of the transcription start site of sex-biased genes in each tissue**. A list of enriched transcription factors of the sex-biased genes in each tissue.

Table S6 **Clinical and lifestyle factors supplied by each data set**. A table representing which data set supplied sample information such as age, ethnicity, sex, smoking status, and disease status.

Table S7 **Total number of sex-biased genes at different log_2_FC cut-offs**. A table listing the total number of genes that were found to be sex-biased in each tissue at different log_2_FC cut-offs. This analysis was performed with the sex chromosomes included.

Figure S1 **Venn diagrams representing the overlap of defined sex-biased genes between this study and a previous study (Melé et al., 2015)**. Each Venn diagram represents an individual tissue and the overlap of genes that were found to be sex-biased between studies.

File S1 **Detailed methodology**. A description of the precise methods used involved in data collection, data processing, normalization, batch correction, and differential expression.
