# Supplementary figures and images for: Large Scale Gene Expression Meta-Analysis Reveals Tissue-Specific, Sex-Biased Gene Expression in Humans
Source: Front Genet. 2016 Oct 13;7:183. doi: 10.3389/fgene.2016.00183 (PMC5062749; doi:10.3389/fgene.2016.00183)

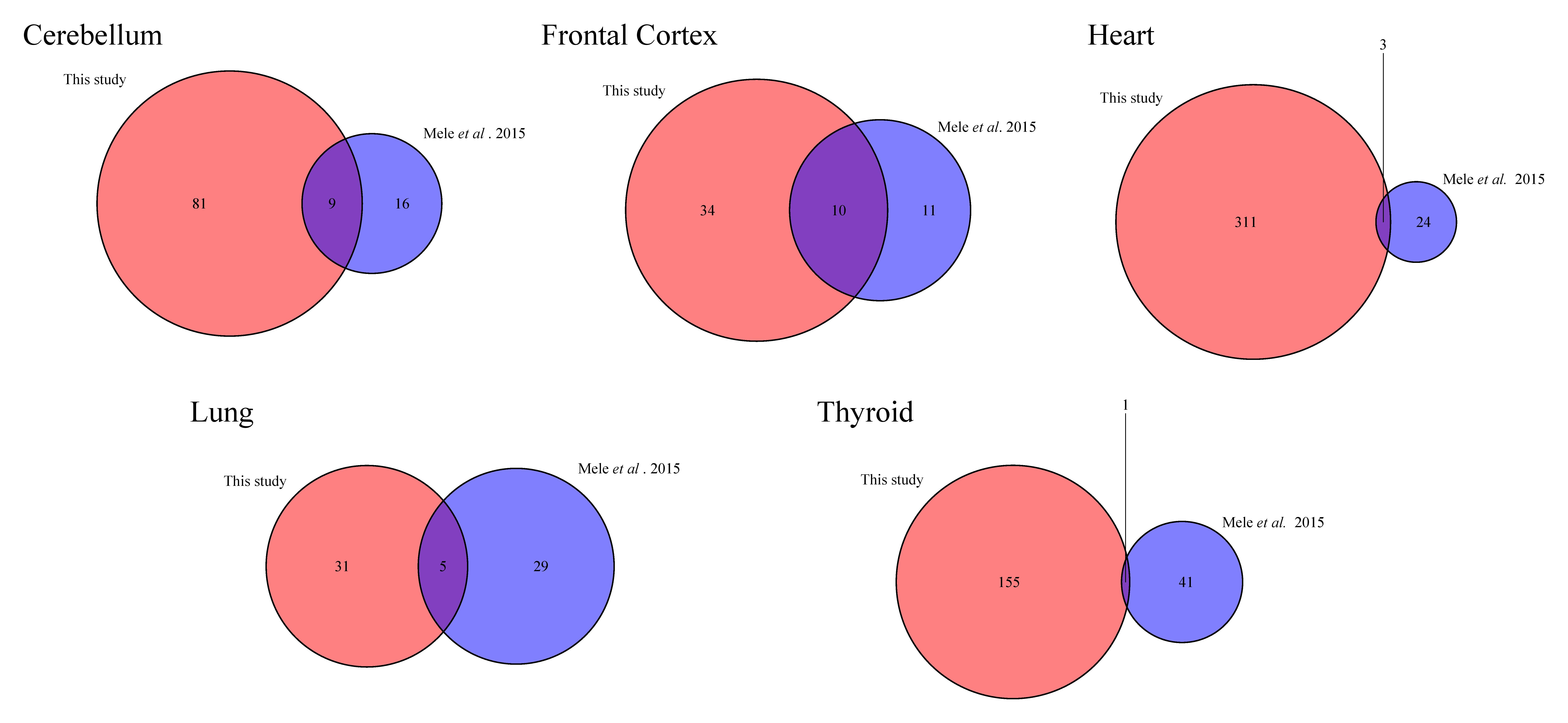

Supplement: Figure S1 — Venn diagrams representing the overlap of defined sex-biased genes between this study and a previous study (Melé et al., 2015). Each Venn diagram represents an individual tissue and the overlap of genes that were found to be sex-biased between studies. [file Image1.JPEG]
