# Supplementary material for: Large Scale Gene Expression Meta-Analysis Reveals Tissue-Specific, Sex-Biased Gene Expression in Humans
Source: Front Genet. 2016 Oct 13;7:183. doi: 10.3389/fgene.2016.00183 (PMC5062749; doi:10.3389/fgene.2016.00183)
Supplement: File S1 — Detailed methodology. A description of the precise methods used involved in data collection, data processing, normalization, batch correction, and differential expression. [file DataSheet1.DOCX]

File S1: Additional details for materials and methods for “Large scale gene expression meta-analysis reveals tissue-specific, sex-biased gene expression in humans”

**Overview**

In this additional file we supply more detail on the precise methods used to conduct our gene expression meta-analysis.

**Data collection**

Initially, we searched the Gene Expression Omnibus (GEO) [1] and ArrayExpress [2] for microarray data sets containing human tissues from healthy individuals. We identified 22 microarray data sets containing 15 different human tissues from 9 different organs. Unfortunately, we were unable to consider lifestyle and clinical factors such as smoking, body mass index (BMI), age and ethnicity since most data sets did not supply any of this information (Additional File 8). We accept not being able to correct for these factors is a limitation of our study. However, despite the enormous generosity of the researchers who had made their data publicly available we think researchers should be encouraged to collect and supply clinical data with future genomic data sets. Despite a lack of clinical and lifestyle data on individual samples we were able to select samples from data sets where the individual did not have any cancerous tissue in their body. For example, in the data set GSE61276, there were liver tissue samples from fetuses, adult liver tissues from individuals who had met accidental death and liver tissue from individuals who had malignant tumours. Therefore, we selected only adult liver tissue samples from individuals who had met accidental death. Although the precise details of death and other clinical details of each individual in GSE61276 is unknown to the public, by removing samples from individuals who had malignancies reduced the potential for confounders in our differential expression analyses. In addition and as stated in our manuscript we chose data sets that had more than 10 samples for better sample sex determination which is described in more detail in the ‘Identifying sample sex’ section.

**Data processing and normalisation**

The 22 microarray data sets used in this study used either an Affymetrix or Illumina platform and the pre-processing and normalisation for each platform was tailored by using a variety of Bioconductor packages (<http://bioconductor.org/>). For studies that used Affymetrix platforms, we firstly downloaded the individual .CEL files from GEO and used simpleaffy [3] to generate a matrix of normalised expression values for each individual data sets. In studies that had used Illumina platforms, we used beadarray [4] which too generated a matrix of normalised expression values for data sets using Illumina platforms.

As detailed in Additional File 6, 4 of the 22 data sets contained data obtained in batches. To correct for these batch effects in these three individual data sets we used the Combat function in the SVA package [5].

**Differential expression analyses**

Differential expression analysis between males and females was performed independently in each tissue. Depending on the number of data sets used in each tissue we used either one of two methods to identify sex-biased genes.

For tissues such as the bladder, where there was only one available data set we used the Empirical Bayes method that is described within the limma package [6]. For tissues such as the heart, where we had more than one data set containing the tissue we used *meta*GEM package (<https://spiral.imperial.ac.uk/handle/10044/1/4217>) and used the inverse–variance method as previously described [7]. We chose the *meta*GEM approach with tissues with more than one data set since the data set have used different microarray platforms. In other words, since each microarray platform has used different probes to annotate for gene expression, their expression values cannot be grouped together. Furthermore, using this approach allows for correction of the data set size using Hedges’ g. Using this approach allows the use of more samples that have been analysed on multiple different microarray platforms which is beneficial to gaining sufficient statistical power.

One of the limitations of using these two approaches is that the magnitude difference in gene expression between males and females is not necessarily interchangeable. To overcome this limitation, one approach would be to use a single platform to measure gene expression such as RNA sequencing. However, there are currently many more publicly available samples that have been analysed by microarrays than by RNA-seq. We therefore focused on using microarrays in this study to maximise the statistical power of our analyses.

**References**

1. Barrett T, Wilhite SE, Ledoux P, Evangelista C, Kim IF, Tomashevsky M, Marshall KA, Phillippy KH, Sherman PM, Holko M, et al: **NCBI GEO: archive for functional genomics data sets--update.** *Nucleic Acids Res* 2013, **41:**D991-995.

2. Brazma A, Parkinson H, Sarkans U, Shojatalab M, Vilo J, Abeygunawardena N, Holloway E, Kapushesky M, Kemmeren P, Lara GG, et al: **ArrayExpress—a public repository for microarray gene expression data at the EBI.** *Nucleic Acids Research* 2003, **31:**68-71.

3. Wilson CL, Miller CJ: **Simpleaffy: a BioConductor package for Affymetrix Quality Control and data analysis.** *Bioinformatics* 2005, **21:**3683-3685.

4. Dunning MJ, Smith ML, Ritchie ME, Tavare S: **beadarray: R classes and methods for Illumina bead-based data.** *Bioinformatics* 2007, **23:**2183-2184.

5. Leek JT, Johnson WE, Parker HS, Jaffe AE, Storey JD: **The sva package for removing batch effects and other unwanted variation in high-throughput experiments.** *Bioinformatics* 2012, **28:**882-883.

6. Ritchie ME, Phipson B, Wu D, Hu Y, Law CW, Shi W, Smyth GK: **limma powers differential expression analyses for RNA-sequencing and microarray studies.** *Nucleic Acids Res* 2015.

7. Ramasamy A, Mondry A, Holmes CC, Altman DG: **Key issues in conducting a meta-analysis of gene expression microarray datasets.** *PLoS Med* 2008, **5:**e184.
